# *Streptococcus pyogenes* Colonization in Children Aged 24–59 Months in the Gambia: Impact of Live Attenuated Influenza Vaccine and Associated Serological Responses

**DOI:** 10.1093/infdis/jiad153

**Published:** 2023-05-29

**Authors:** Alexander J Keeley, Danielle Groves, Edwin P Armitage, Elina Senghore, Ya Jankey Jagne, Hadijatou J Sallah, Sainabou Drammeh, Adri Angyal, Hailey Hornsby, Gabrielle de Crombrugghe, Pierre R Smeesters, Omar Rossi, Martina Carducci, Chikondi Peno, Debby Bogaert, Beate Kampmann, Michael Marks, Helen A Shaw, Claire R Turner, Thushan I de Silva

**Affiliations:** Faculty of Infectious and Tropical Diseases, London School of Hygiene and Tropical Medicine, London, United Kingdom; Department of Infection, Immunity, and Cardiovascular Disease, Medical School, University of Sheffield, Sheffield, United Kingdom; Vaccines and Immunity Theme, Medical Research Council Unit The Gambia at London School of Hygiene and Tropical Medicine, Banjul, The Gambia; Department of Infection, Immunity, and Cardiovascular Disease, Medical School, University of Sheffield, Sheffield, United Kingdom; Faculty of Infectious and Tropical Diseases, London School of Hygiene and Tropical Medicine, London, United Kingdom; Vaccines and Immunity Theme, Medical Research Council Unit The Gambia at London School of Hygiene and Tropical Medicine, Banjul, The Gambia; Vaccines and Immunity Theme, Medical Research Council Unit The Gambia at London School of Hygiene and Tropical Medicine, Banjul, The Gambia; Vaccines and Immunity Theme, Medical Research Council Unit The Gambia at London School of Hygiene and Tropical Medicine, Banjul, The Gambia; Vaccines and Immunity Theme, Medical Research Council Unit The Gambia at London School of Hygiene and Tropical Medicine, Banjul, The Gambia; Vaccines and Immunity Theme, Medical Research Council Unit The Gambia at London School of Hygiene and Tropical Medicine, Banjul, The Gambia; Department of Infection, Immunity, and Cardiovascular Disease, Medical School, University of Sheffield, Sheffield, United Kingdom; Department of Infection, Immunity, and Cardiovascular Disease, Medical School, University of Sheffield, Sheffield, United Kingdom; Molecular Bacteriology Laboratory, Université Libre de Bruxelles, Brussels, Belgium; Department of Pediatrics, Hôpital Universitaire des Enfants Reine Fabiola, Université Libre de Bruxelles Brussels, Belgium; Molecular Bacteriology Laboratory, Université Libre de Bruxelles, Brussels, Belgium; Department of Pediatrics, Hôpital Universitaire des Enfants Reine Fabiola, Université Libre de Bruxelles Brussels, Belgium; GSK Vaccines Institute for Global Health, Siena, Italy; GSK Vaccines Institute for Global Health, Siena, Italy; Centre for Inflammation Research, Queen's Medical Research Institute, University of Edinburgh, Edinburgh, United Kingdom; Centre for Inflammation Research, Queen's Medical Research Institute, University of Edinburgh, Edinburgh, United Kingdom; Faculty of Infectious and Tropical Diseases, London School of Hygiene and Tropical Medicine, London, United Kingdom; Vaccines and Immunity Theme, Medical Research Council Unit The Gambia at London School of Hygiene and Tropical Medicine, Banjul, The Gambia; Charité Centre for Global Health and Institut für Internationale Gesundheit, Charité - Universitätsmedizin Berlin, Berlin, Germany; Faculty of Infectious and Tropical Diseases, London School of Hygiene and Tropical Medicine, London, United Kingdom; Hospital for Tropical Diseases, University College London Hospital, London, United Kingdom; Division of Infection and Immunity, University College London, London, United Kingdom; Vaccines Division, Scientific Research and Innovation Group, Medicines and Healthcare Products Regulatory Agency, Potters Bar, United Kingdom; School of Biosciences, University of Sheffield, Sheffield, United Kingdom; Faculty of Infectious and Tropical Diseases, London School of Hygiene and Tropical Medicine, London, United Kingdom; Department of Infection, Immunity, and Cardiovascular Disease, Medical School, University of Sheffield, Sheffield, United Kingdom; Vaccines and Immunity Theme, Medical Research Council Unit The Gambia at London School of Hygiene and Tropical Medicine, Banjul, The Gambia

**Keywords:** antibodies, carriage, colonization, live attenuated influenza vaccine, serological responses, *streptococcus pyogenes*, The Gambia

## Abstract

**Background:**

Immunity to *Streptococcus pyogenes* in high burden settings is poorly understood. We explored *S. pyogenes* nasopharyngeal colonization after intranasal live attenuated influenza vaccine (LAIV) among Gambian children aged 24–59 months, and resulting serological response to 7 antigens.

**Methods:**

A post hoc analysis was performed in 320 children randomized to receive LAIV at baseline (LAIV group) or not (control). *S. pyogenes* colonization was determined by quantitative polymerase chain reaction (qPCR) on nasopharyngeal swabs from baseline (day 0), day 7, and day 21. Anti-streptococcal IgG was quantified, including a subset with paired serum before/after *S. pyogenes* acquisition.

**Results:**

The point prevalence of *S. pyogenes* colonization was 7%–13%. In children negative at day 0, *S. pyogenes* was detected at day 7 or 21 in 18% of LAIV group and 11% of control group participants (*P* = .12). The odds ratio (OR) for colonization over time was significantly increased in the LAIV group (day 21 vs day 0 OR, 3.18; *P* = .003) but not in the control group (OR, 0.86; *P* = .79). The highest IgG increases following asymptomatic colonization were seen for M1 and SpyCEP proteins.

**Conclusions:**

Asymptomatic *S. pyogenes* colonization appears modestly increased by LAIV, and may be immunologically significant. LAIV could be used to study influenza-*S. pyogenes* interactions.

**Clinical Trials Registration**. NCT02972957.

## Teaser

Understanding natural immunity to *Streptococcus pyogenes* is essential towards a safe and effective vaccine. During 21-day follow-up, 22% of Gambian children aged 24–59 months were colonized with *S. pyogenes.* Colonization provoked serological responses, particularly to M1 and SpyCEP antigens.

*Streptococcus pyogenes* (group A *Streptococcus*, Strep A) is responsible for half a million deaths worldwide each year, mainly in low- and middle-income countries (LMIC), through severe invasive infections and immune complications including rheumatic heart disease [1, 2]. Development of a vaccine against *S. pyogenes* was declared a global health research priority in 2018 by the World Health Assembly. An ideal vaccine would substantially reduce *S. pyogenes* transmission, disease, and perhaps colonization, without provoking immune-mediated complications [3]. Observational data demonstrate decreased *S. pyogenes* incidence with increasing age, possibly explained by naturally acquired immunity [4]. Estimated point prevalence of asymptomatic pharyngeal colonization with *S. pyogene*s is 6.6%–9.7% in children [4]. Characterizing protective immunity generated by infections and colonization remains a major knowledge gap in designing effective and safe vaccines, particularly from LMIC settings [5–7].

Epidemiological observations have demonstrated association between invasive *S. pyogenes* infections and respiratory viral infections, especially influenza [8]. *S. pyogenes* was responsible for substantial mortality in influenza pandemics of 1918 and 2009 [8, 9]. It is important to understand whether respiratory viruses increase colonization with pathogenic bacteria such as *S. pyogenes* in the nasopharynx, as colonization may be a necessary preceding event to pharyngitis and invasive disease. We have recently demonstrated that intranasal live attenuated influenza vaccine (LAIV), used as a surrogate viral challenge agent, induces modest increases in *Streptococcus pneumoniae* colonization and density in the 21 days following vaccination in Gambian children [10]. We conducted a post hoc analysis of this randomized controlled trial of LAIV in children aged 24–59 months to explore whether LAIV increased nasopharyngeal *S. pyogenes* colonization, and whether colonization was associated with a serological response to several *S. pyogenes* antigens.

## METHODS

### Study Population and Design

We conducted a post hoc observational study nested within a randomized controlled trial of LAIV, studying immunogenicity, viral shedding and microbiome interactions in children aged 24–59 months in Sukauta, an urban region of The Gambia. The study (NCT02972957) was conducted over 2 years between February to April 2017, and January to March 2018 [10, 11]. Children were randomized 2:1 to receive LAIV at study entry (day 0, LAIV group) or on day 21 (control group), which was the end of active follow-up. All participants were influenza-vaccine naive and clinically well, with no history of respiratory illness in the prior 14 days. Participants in LAIV and control groups were recruited simultaneously to avoid bias via seasonal variation. Baseline respiratory virus status was determined with multiplex polymerase chain reaction (PCR) [10]. Children were immunized with the Northern Hemisphere Russian-backbone Trivalent LAIV (Nasovac-S, Serum Institute of India, Pune, India) [11]. Data on symptoms experienced between days 0 and 7 were collected at the day 7 visit, and symptoms experienced between days 7 and 21 recorded at the day 21 visit. The study was approved by the Joint Medical Research Council/Gambia Government ethics committee (reference 16193). Informed consent was obtained from all parents, including for subsequent research on their samples.

### Determination of *S*. *pyogenes* Colonization Status

**Figure 1.**
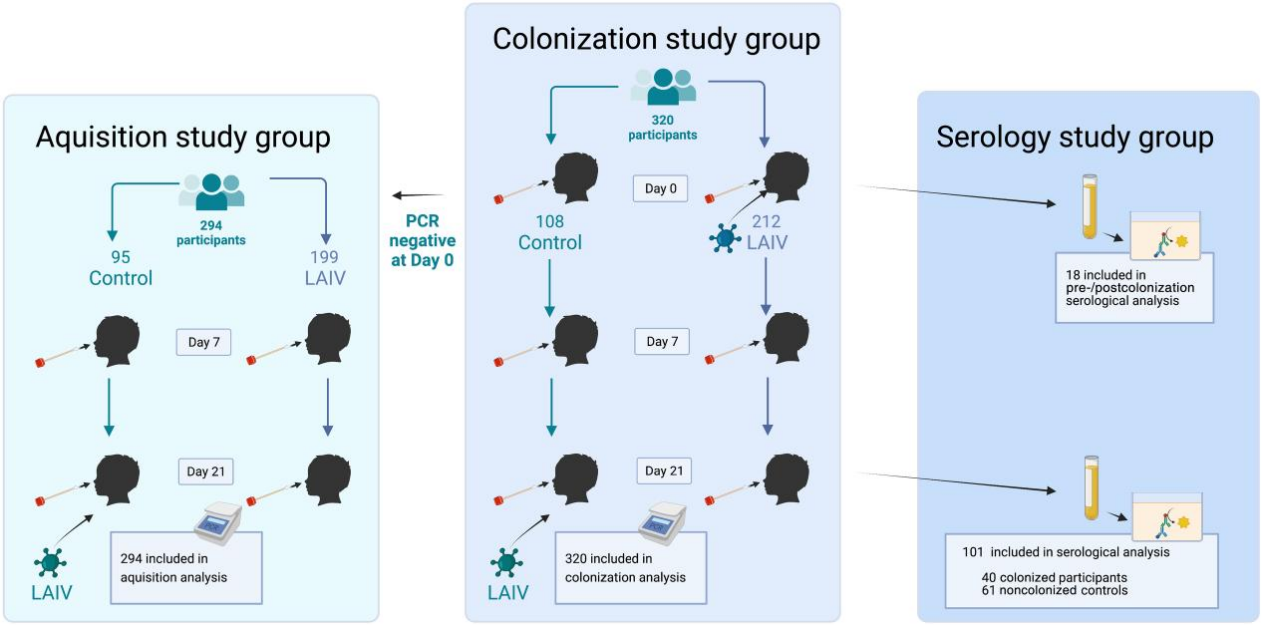
Study profile: 320 participants were randomized 2:1 to the LAIV (day 0 vaccine) or control (day 21 vaccine) group. All participants (colonization study group) had *Streptococcus pyogenes* colonization status determined by quantitative PCR at day 0, 7, and 21. Of the 320 participants, 294 were negative at baseline (the acquisition study group). Only participants receiving LAIV at day 0 had serum taken at days 0 and 21 (n = 212). Serum from day 21 was available from 40/48 participants from the LAIV group who were colonized at any time point and from 61/164 randomly selected noncolonized participants. Paired serum (pre-/postcolonization) was available from 18/35 participants who acquired colonization during the study period. Created with BioRender.com. Abbreviations: LAIV, live attenuated influenza vaccine; PCR, polymerase chain reaction.

Participants had a nasopharyngeal swab taken at day 0 (immediately prior to LAIV receipt), days 7 and 21, using flocked swabs (FLOQSwabs) stored in RNAprotect (Qiagen). Samples were processed within 4 hours of collection and stored at −70°C until further processing. DNA was extracted from 200 μL of RNAprotect using the AGOWA Mag Mini DNA extraction kit (LGC Genomics) in combination with phenol-bead beating as previously described [12]. Standard curves were generated via extraction of total genomic DNA from the *S. pyogenes* H293 reference strain, ranging from 1 to 10 000 000 genome copies per μL. Quantitative PCR was performed using QuantStudio 5 Real-Time PCR System, using primers and probes to detect the *S. pyogenes-*specific gene *speB* (forward, CTAAACCCTTCAGCTCTTGGTACTG; reverse, TTGATGCCTACAACAGCACTTTG, Probe: Cy5-CGGCGCAGGCGGCTTCAAC-BHQ2) [13]. A cycle threshold (Ct) of 40 was defined as positive, but all curves above Ct threshold of 35 were checked manually to ensure appearance was consistent with true amplification of the target. Samples were run in triplicate. Where only 1 of the triplicates showed the presence of *S. pyogenes*, samples were repeated and only defined as positive if at least 2 triplicates were positive on repeat testing. A participant with positive quantitative PCR (qPCR) result at a given time point was considered colonized. All participants were included in the colonization study group. Participants who were negative at baseline were included in the acquisition study group (Figure 1).

### Antibody Measurement

Blood was collected in serum separation tubes from participants in the LAIV group, for study end points on influenza vaccine immunogenicity [11]. For the serology study, participants were categorized as colonized if *S. pyogenes* was detected by qPCR at any time point. Participants in the acquisition study group (negative at day 0) were categorized as newly colonized if *S. pyogenes* was detected at either day 7 or 21. All available serum collected on day 21 from colonized participants was tested, along with a random selection from noncolonized participants (a 1:1.5 ratio of colonized to noncolonized participants). Of newly colonized participants, a subgroup of 18 had paired serum (pre-/postcolonization) available for testing.

Enzyme-linked immunosorbent assay (ELISA) optimization was performed to determine optimal *S. pyogenes* protein coating concentrations, blocking buffer solution, serum dilution, and secondary antibody concentration. Ninety-six–well flat-bottomed high-binding ELISA plates were coated overnight with several recombinant proteins in pH 9.6 carbonate buffer: *S. pyogenes* cell envelope protease (SpyCEP) at 0.16 μg/mL, *S. pyogenes* adhesion and division protein (SpyAD) at 1 μg/mL, M1 protein at 0.5 μg/mL, Mac/IdeS protein (henceforth referred to as Mac) at 1 μg/mL, and collagen binding protein (Cpa) at 0.16 μg/mL. Proteins were provided by National Institute for Biological Standards and Control, Medicines and Healthcare Products Regulatory Agency, UK and derived from the reference SF370 strain (M1/*emm*1/FCT-2) (Supplementary Material) [14]. Blocking and serum dilution was performed using 1% casein blocking buffer (ThermoFisher Scientific). Serum was diluted to 1:100 for Cpa and Mac, and 1:800 for M1, SpyCEP, and SpyAD, and applied to protein-coated wells. Bound immunoglobulin G (IgG) was detected with goat anti-human IgG-horseradish peroxidase conjugate (Invitrogen), diluted to 1:500, then incubated with SureBlue 3,3',5,5'-tetramethylbenzidine (TMB) 1-Component Peroxidase Substrate (KPL). The reaction was stopped with 1% hydrochloric acid and optical density (OD) was read at 450 nm, subtracting background OD. Six washes were performed between each ELISA step with 0.05% Tween-20 in phosphate-buffered saline. To quantify anti-protein IgG activity in participant sera, 12 serial 3-fold dilutions of pooled human immunoglobulin (Gammanorm, Octapharm) were used to generate standard curves commencing at 1:33.3 dilution (14.85 mg/mL) in 1% casein buffer. Seroconversion between days 0 and 21 was considered as a 2-fold rise in antibody titer, or 4-fold rise in antibody titer if raw optical density measurements were <0.25 at baseline, as per previously published definitions [15].

To measure antibody titers to the additional vaccine antigens group A carbohydrate (GAC) and streptolysin O (SLO), an optimized 4-plex (GAC, SLO, SpyCEP, SpyAD) serology assay using the Luminex platform was performed on all available sera using a described protocol [16]. All antigens were supplied by GSK Vaccine Institute for Global Health. All sera with ELISA data were tested, except 2 samples from noncolonized participants where there was no remaining volume. Samples were tested in duplicate at a dilution of 1:8100 alongside standard curves derived from pooled intravenous immunoglobulin (IVIG; Privigen, CSL Behring). Mean florescence intensity was measured for each antigen with a Luminex 200 instrument (Invitrogen).

### Statistical Analysis

All statistical analysis was performed in R (version 4.0.1). Comparison of *S. pyogenes* colonization status and incident acquisition between children who received LAIV and those who did not was performed using χ^2^ test. In addition, logistic and generalized mixed effects logistic regression models, accounting for multiple time point sampling from individuals, were used to explore the change in colonization status over time within the vaccinated and unvaccinated groups, as previously described [10]. Covariates included were age in months, sex, the presence of asymptomatic respiratory viruses at baseline, time point (day 0 vs day 7, day 0 vs day 21) and receipt of LAIV [10, 11].

Samples where optical density measured by ELISA fell below the detection limit of the standard curve, were allocated a random value between zero and the limit of detection. Antibody titers in Luminex data were interpolated using xPonent 4.2 software (Luminex Corporation) using 5-parameter logistic regression. For the 4-plex Luminex assay, where both limit of accurate quantification and detection were characterized, results falling below respective limits were randomly assigned a value between zero and the lowest value of each limit [16]. Antibody data (IVIG-adjusted anti-protein activity in mg/mL for ELISA and relative Luminex units [RLU/mL] for Luminex assay) were log transformed and assessed for normality using QQplot and the Shapiro-Wilk test. Log transformed antibody quantities between groups were compared using Student *t* test. Paired Student *t* test was performed to compare antibody levels before and after *S. pyogenes* colonization from individual participants. A *P* value of <.05 was considered statistically significant. Correlation between log transformed antibody titers to different antigens within individuals was assessed with the Spearman method.

## RESULTS

### *S. pyogenes* Colonization Prevalence and Incidence

A total of 320 participants were included in this study, of which 212 received LAIV (Figure 1). Overall, 71/320 (22%) children were colonized with *S. pyogenes* on at least 1 time point within the 21-day study period. At day 0, 26/320 (8%) participants were colonized, 13 in the LAIV (6%) and 13 in control (12%) groups (*P* = .068; Supplementary Table 1), and 109/320 (34%) participants had a baseline respiratory virus detected at day 0. There was no difference in colonization at baseline between participants with a detectable respiratory virus and those without (11/109, 10% vs 15/211, 7.1%, *P* = .4). In the acquisition study group, 45 (15%) had acquired colonization by day 7 or 21 (Table 1), 35/199 (18%) in the LAIV group, and 10/95 (11%) in the control group (Table 1, *P* = .12). A logistic regression model was used to explore the odds of new *S. pyogenes* colonization at either day 7 or 21 in the acquisition study group (n = 294), accounting for age, sex, LAIV receipt, and the presence of other respiratory viruses at day 0 (Supplementary Table 2), showing no significant association between LAIV and *S. pyogenes* acquisition (odds ratio [OR], 1.92; 95% confidence interval [CI], .93–4.31; *P* = .09).

**Table 1.** *Streptococcus pyogenes* Acquisition During the 21-Day Study Period in the Acquisition Study Group

*Time* Point	*N*o.	*Overall,* n = *294*	*Control,* n = *95*	*LAIV,* n = *199*	*P* Value^a^
*Day 7*	294	18 (6)	3 (3)	15 (8)	.14
*Day 21*	294	35 (12)	8 (8)	27 (14)	.2
*Any time*	294	45 (15)	10 (11)	35 (18)	.12

Data are No. (%).

Pearson χ^2^ test.

To explore changes in colonization status within an individual over time in all children (n = 320), a generalized logistic mixed effects regression model was used to explore the odds of *S. pyogenes* colonization over time, accounting for age, sex, LAIV receipt, and the presence of other respiratory viruses at day 0 (Supplementary Table 3). This model demonstrated a significant interaction between LAIV receipt and the day 21 time point terms. Therefore we constructed separate models for the LAIV and control groups. The OR of *S. pyogenes* colonization was higher at day 21 compared to day 0 in the LAIV group (OR, 3.18; 95% CI, 1.49–6.81; *P* = .003; Table 2) but not day 7 (OR, 1.30; 95% CI, .57–2.97; *P* = .54). The OR of *S. pyogenes* colonization in the control group was not higher at either day 7 (OR, 0.34; 95% CI, .09–1.27; *P* = .11) or day 21 (OR, 0.86; 95% CI, .3–2.51; *P* = .79) when compared to day 0 (Supplementary Table 4).

**Table 2.** Factors Associated With *Streptococcus pyogenes* Colonization in the Live Attenuated Influenza Vaccine Group (n = 212)

	OR	95% CI	*P* Value
Day 7 vs day 0	1.30	.57–2.97	.540
Day 21 vs day 0	3.18	1.49–6.81	.**003**
Positive respiratory virus at day 0	1.54	.72–3.29	.266
Age in months	0.97	.93–1.01	.162
Sex male vs female	0.97	.47–2.04	.945

*P* values for factors associated with *Streptococcus pyogenes* colonization are derived from a generalized logistic mixed effects model, taking into account changes within individuals over time. Bold signifies a *P* value of <0.05 considered statistically significant.

Abbreviations: CI, confidence interval; OR, odds ratio.

Only 17 of 71 (23.9%) participants with *S. pyogenes* detected during the study were colonized at 2 time points, with only 2 (2.8%) colonized at all 3 study time points (Supplementary Figure 1). No difference in qPCR-quantified *S. pyogenes* density was observed between LAIV and control participants at any time point, nor in episodes with persistent positivity at subsequent study visits (Supplementary Table 5A and 5B).

We compared symptom data during the study period for all 294 children in the acquisition study group. Ten children had infected skin sores during the 21-day follow-up, 9 in the LAIV group and 1 in the control group (5% vs 1%, *P* = .2; Supplementary Table 6). Only 3 episodes of sore throat were reported, all in the LAIV group (Supplementary Table 6). Skin sores were more common in those who became colonized (9% vs 2%, *P* = .05; Supplementary Table 6). No statistically significant difference in fever, cough, rhinorrhea, or sore throat was seen in children who acquired *S. pyogenes* compared to those who did not (Supplementary Table 6). In the total study group only 6/71 (9%) colonized participants had either a sore throat or an infected skin sore.

### Serological Responses to *S*. *pyogenes* Antigens

**Figure 2.**
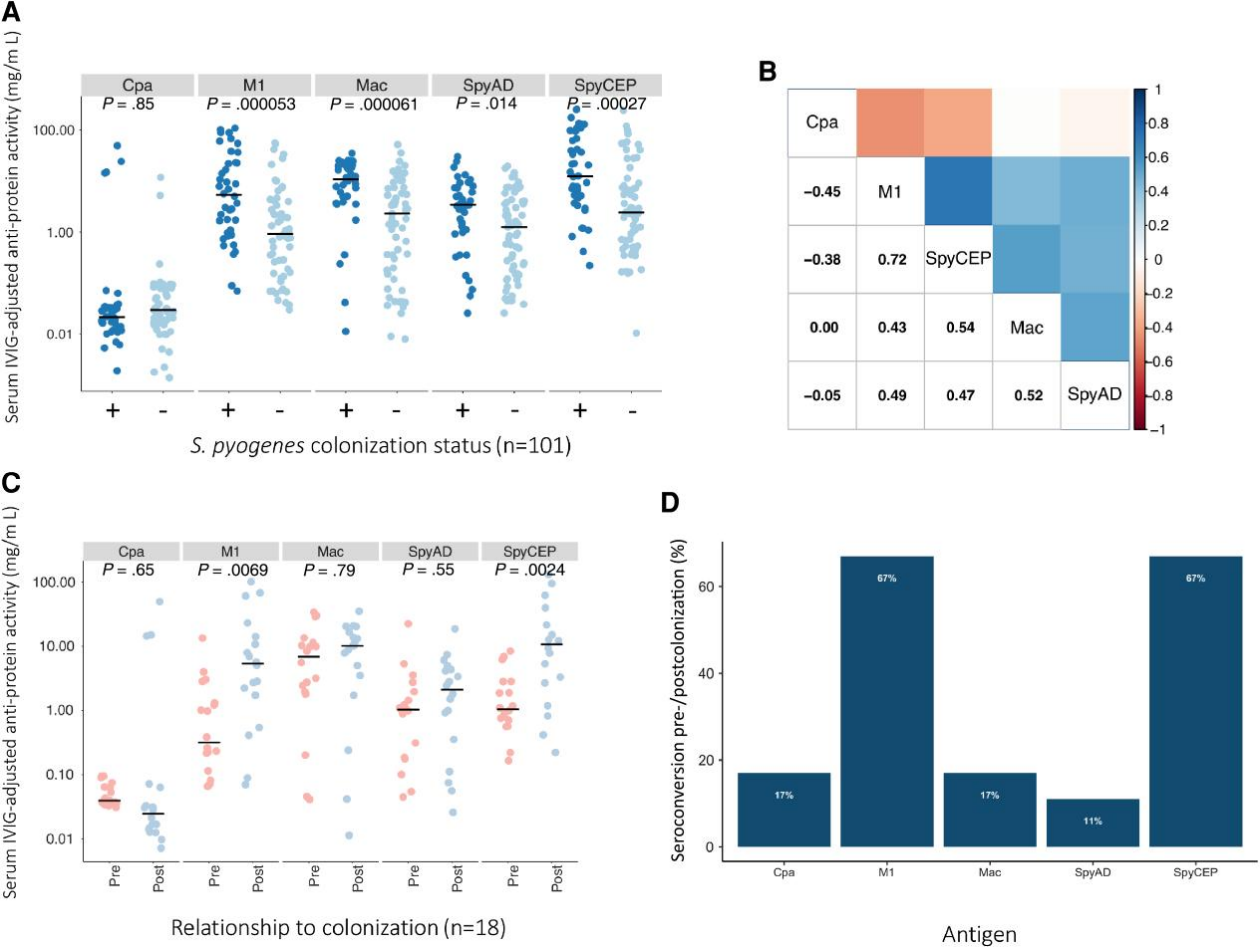
Serological responses to *Streptococcus pyogenes* colonization measured by ELISA. *A*, Comparison of anti-protein IgG activity to Cpa, M1, Mac, SpyCEP, and SpyAD in participants (n = 101) according to anytime *S. pyogenes* colonization status. Log^10^ transformed IVIG-adjusted anti-protein activity was compared between colonized and uncolonized participants with unpaired *t* tests; horizontal line depicts the median value. *B*, Pairwise correlation coefficients (Spearman method) of IgG titer measured by ELISA within individual participants (n = 101). *C*, Paired comparison of anti-protein IgG activity to Cpa, M1, Mac, SpyCEP, and SpyAD between day 0 and day 21 in newly colonized participants (n = 18). Log_10_ transformed IVIG-adjusted anti-protein activity was compared between pre and post colonization samples with paired *t*-tests; horizontal line depicts the median value. *D*, Percentage of study participants (n = 18) acquiring *S. pyogenes* during the study who seroconverted between day 0 and day 21. Abbreviations: ELISA, enzyme-linked immunosorbent assay; IgG, immunoglobulin G; IVIG, intravenous immunoglobulin.

**Figure 3.**
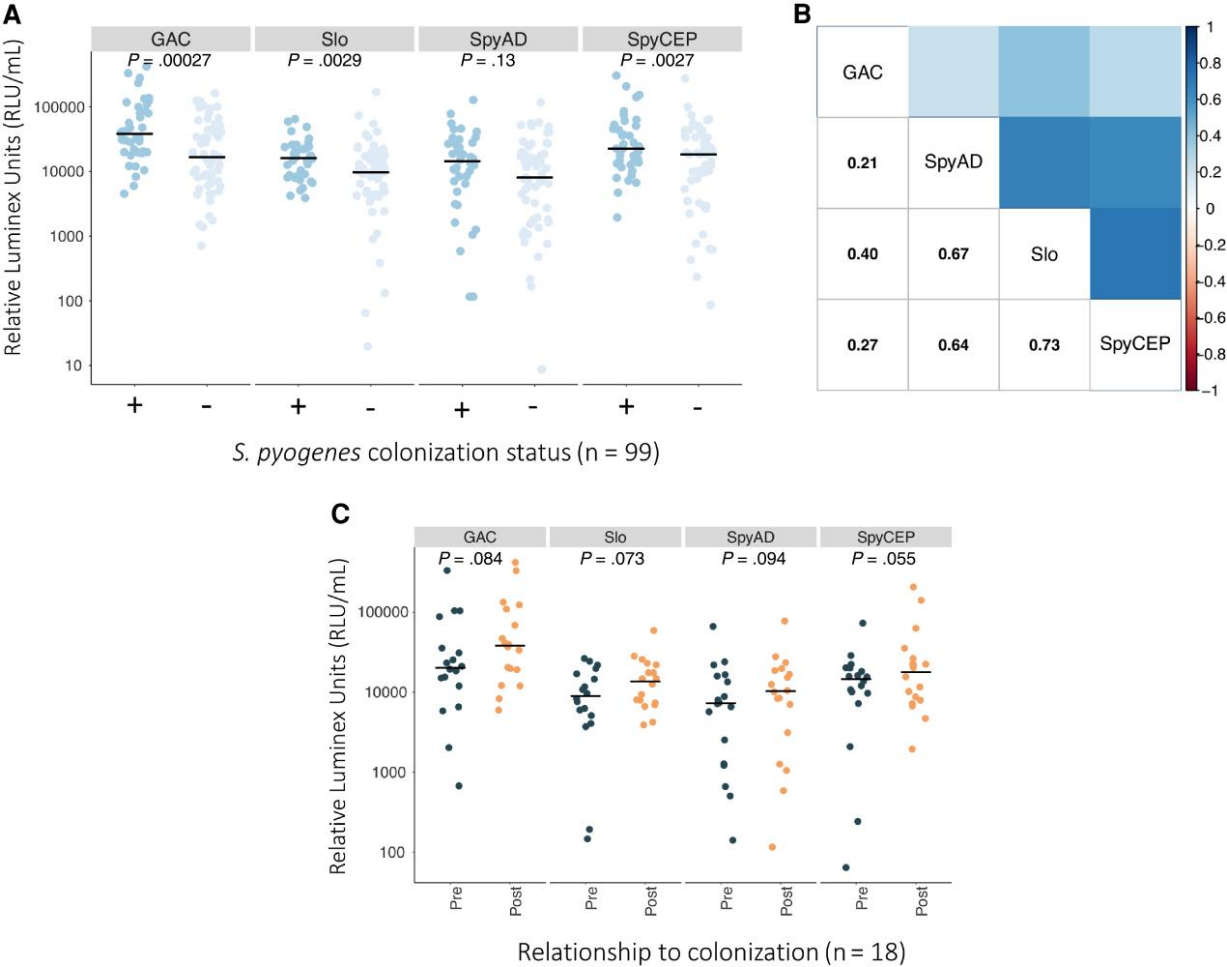
Serological responses to *Streptococcus pyogenes* colonization measured by Luminex 4-plex. *A*, Comparison of IgG activity to GAC, SLO, SpyCEP, and SpyAD in participants (n = 99) according to anytime *S. pyogenes* colonization status. *B*, Pairwise correlation coefficients (Spearman method) of IgG titer measured by Luminex within individual participants (n = 99). *C*, Paired comparison of IgG activity to GAC, SLO, SpyCEP, and SpyAD between days 0 and 21 in newly colonized participants (n = 18). Log_10_ transformed IVIG-adjusted anti-antigen activity was compared with *t* tests (unpaired and paired, accordingly). Horizontal bar depicts the median value. Abbreviations: IgG, immunoglobulin G; IVIG, intravenous immunoglobulin.

ELISA-quantified serum antibody titers at day 21 in 40/48 colonized participants were compared with those in 61 randomly selected noncolonized participants (all from the LAIV group due to sera availability). Missing serum was due to insufficient remaining material from the parent study. The age was not significantly different between participants included in serological study who were colonized compared to noncolonized controls (median age in months 36 vs 32, *P* = .3; Supplementary Table 7). Sera from colonized participants demonstrated significantly higher IVIG-adjusted IgG levels to M1, SpyCEP, SpyAD, and Mac, but not Cpa, compared to noncolonized participants (Figure 2*A*). Within individuals the strongest correlation was observed between IgG titers for M1 and for SpyCEP (Figure 2*B*). Paired serum was available for 18/35 newly colonized participants. Mean M1- and SpyCEP-specific IgG titers were significantly increased at day 21 compared to day 0, but not IgG titers to SpyAD, Mac, or Cpa (Figure 2*C*). The proportion of newly colonized participants who seroconverted pre-/postcolonization was greatest for M1 and SpyCEP (Figure 2*D*). To explore serological responses further, a recently described 4-plex assay was used to quantify antibodies to additional *S. pyogenes* vaccine antigens GAC and SLO, along with SpyCEP and SpyAD. This assay was tested on 99/101 samples with ELISA-quantified anti-*S. pyogenes* titers [16]. Sera from colonized children demonstrated significantly higher titers to GAC, SLO, and SpyCEP but not to SpyAD (Figure 3*A*). Within individuals the strongest correlation was observed between IgG titers for SLO and for SpyCEP (Figure 3*B*). In the subset of participants with *S. pyogenes* acquisition during the 21-day study period (n = 18), differences in IgG titers at days 0 and 21 were not statistically significant (Figure 3*C*). Antibody titers to SpyCEP and SpyAD that were measured by both techniques were well correlated (Supplementary Figure 2). Removing the participants with sore throat or infected skin sores and colonization during the study had no significant impact on the serological results, nor did excluding participants colonized on day 21 only, nor applying a more conservative definition of seroconversion (4-fold increase in IgG titer regardless of baseline optical density) (Supplementary Figures 3, 4, and 5).

## DISCUSSION

In this post hoc study of a randomized controlled trial in The Gambia, we analyzed the impact of LAIV on *S. pyogenes* nasopharyngeal colonization in children aged 24–59 months. Using PCR to define colonization events, we observed an 8% prevalence at study baseline. Our data demonstrate only a modest impact of LAIV on colonization rates. We observed nonsignificantly increased rates and odds of new colonization in the LAIV group and increased odds of colonization at day 21 compared to baseline in the LAIV group only. During the 21-day study, 22% of children were colonized at 1 or more time points, with most (73%) only colonized once. The dynamic picture of colonization we observed may be partially influenced by LAIV administration and reflects the common exposure of children to *S. pyogene*s in areas with a high burden of disease. Our findings are consistent with published estimates from LMICs [4, 17].

*S. pyogenes* was responsible for substantial mortality in influenza pandemics of 1918 and 2009 [8, 9]. The impact of respiratory viruses on pharyngeal colonization, modulation of host-pathogen interaction, and promoting severe *S. pyogenes* disease is complex and poorly understood [8, 9, 18–21]. While mice administered with a nonlethal challenge with *S. pyogenes* following an influenza challenge frequently had a severe and fatal disease course [21], mice vaccinated with LAIV were protected from *S. pyogenes* superinfection [20]. Few models have specifically investigated the impact of respiratory viruses on pharyngeal colonization [22]. Through the *S. pyogenes* human challenge model, cochallenge studies with LAIV could reveal mechanistic insights into the interaction between influenza virus, *S. pyogenes*, and host immune responses [23]. A cochallenge study with *S. pneumoniae* and LAIV has shown how virus-induced inflammatory responses and impaired innate immune responses promote bacterial colonization [24]. Whilst no impact on colonization density was demonstrated in this study, it is possible that the observed rates of acquisition in the LAIV group was due to modestly increased colonization density, reaching a threshold for PCR positivity. This mechanism has been observed for other bacteria including *S. pneumoniae,* both in this study population and in other settings [10, 25]. Our data provide further evidence that the impact of LAIV on colonization with potentially pathogenic bacteria is only modest and supports the wider rollout of LAIV to reduce influenza disease and its complications in LMICs.

We also demonstrate that asymptomatic *S. pyogenes* colonization leads to seroconversion to several antigens representative of different stages of *S. pyogenes* infection [14], with higher responses observed in colonized children compared to noncolonized controls. Given that prior exposure to *S. pyogenes* in all children in this cohort would be similar, these differences likely reflect recent exposure. The most notable responses were seen to full-length streptococcal M1 protein and to the envelope protease SpyCEP. Reactivity to full-length M1 protein likely reflects activity to conserved M protein regions rather than type-specific reactivity, given that *emm*1 isolates have not been identified in The Gambia [26, 27]. IgG antibodies to other antigens, SpyAD, Mac, GAC, and SLO, were higher in children where *S. pyogenes* colonization had been detected, but this was not found for IgG antibodies to Cpa. The Cpa used in our study was originally derived from the M1 FCT-2 strain SF370. Our previous study found no Gambian FCT-2 isolates, although approximately 70% were Cpa-positive FCT-3 or FCT-4 isolates [27]. However, Cpa from FCT-3 or FCT-4 shares only approximately 50% amino acid identity with Cpa from FCT-2, which may explain limited Cpa reactivity in this cohort. Noting that the antigens tested via ELISA and Luminex were obtained from different sources, there was broad concurrence between the 2 serological assays, except that IgG to SpyAD was significantly higher in colonized compared to noncolonized participants when measured by ELISA and not by Luminex. A detailed comparison of the 2 platforms to determine the optimal technique for measurement of IgG to these *S. pyogenes* antigens was not performed. Nonetheless, our findings suggest that asymptomatic pharyngeal colonization may induce an IgG immunological response to multiple antigens.

The definition of true *S. pyogenes* colonization, referring to colonization *without* a serological response, risks oversimplification of a complex and dynamic state influenced by host immunity, bacterial characteristics, and environmental factors. This serologically inactive phenotype has been described with minimal serum antibody response to SLO and anti-DNAse B [7, 28]. Our data show that serological responses following asymptomatic colonization vary across different proteins. Furthermore, in practice, colonization is often defined as an asymptomatic person with detectable *S. pyogenes* without serial serological testing. In detailed longitudinal analyses, seroconversion has been documented following asymptomatic acquisition of *S. pyogenes* in the United States and Egypt, with the highest proportion of seroconversions to type-specific M peptides and SpyAD [15, 29]. In another cohort study in the United States, seroconversion to type-specific M protein frequently occurred following asymptomatic acquisition and conferred protection from homologous strain reinfection [30].

Protection following asymptomatic colonization and *S. pyogenes* intranasal vaccination has been observed, but the responsible mechanisms and serological corelates of protection have not been identified [31–36]. Early prospective observation demonstrated the emergence of acute rheumatic fever following asymptomatic *S. pyogenes* infection [37]. Recent evidence suggests that patients with rheumatic fever have more serological activity to *S. pyogenes* M peptides and conserved antigens than matched controls, including after asymptomatic pharyngeal colonization [38, 39]. Asymptomatic colonization may therefore contribute to pathological immunity in endemic settings and warrants further exploration.

Our study has several key limitations. Firstly, it was a post hoc analysis of a study that was not designed or powered to assess the impact of LAIV on *S. pyogenes* colonization. LAIV was used as a proxy for natural influenza infection and the observations may not reflect the true dynamics of *S. pyogenes* colonization during natural influenza infection. We defined colonization events by PCR and not by gold-standard microbiological culture of *S. pyogenes*. Whilst *S. pyogenes* skin infection in children is common in this setting [40], we did not perform microbiological culture on children reporting infected skin lesions nor sore throats in this study. Nonetheless, exclusion of all participants with either a sore throat or infected skin lesion did not dramatically alter the serological findings. Serum IgG could only be measured in the LAIV group, so no serological comparison between vaccinated and unvaccinated groups was possible. Finally, PCR to a single preserved and specific *S. pyogenes* target does not allow for assessment of *emm*-type specific immunity, which is an historically important consideration for *S. pyogenes* serological activity.

Nonetheless, our study provides several important findings. Understanding both naturally occurring protective immunity and pathological autoimmunity to *S. pyogenes* from settings with the highest disease burden is of paramount importance for progress towards a safe and effective *S. pyogenes* vaccine. Our study adds further evidence that asymptomatic colonization may be immunologically significant, particularly in the context of influenza coinfection. Further research combining longitudinal observation with detailed microbiological and immunological investigation should be prioritized from areas of high disease prevalence to gain a deeper understanding of immunity to this major human pathogen.

## Supplementary Data

Supplementary materials are available at *The Journal of Infectious Diseases* online. Consisting of data provided by the authors to benefit the reader, the posted materials are not copyedited and are the sole responsibility of the authors, so questions or comments should be addressed to the corresponding author.

## Supplementary Material

jiad153_Supplementary_Data

## Notes

***Acknowledgments***. The authors acknowledge and thank the Strep A Study Group at Medical Research Council (MRC) Unit The Gambia at London School of Hygiene and Tropical Medicine (LSHTM), Sona Jabang, Annette Erhart, Saffiatou Darboe, Abdul Karim Sesay, Peggy-Estelle Tiencheu, Saikou Y. Bah, Lamin Jaiteh, Karen Forrest, Anna Roca, Fatoumata Camara; Peggy-Estelle Tiencheu, Aru-Kumba Baldeh, Grant Mackenzie, Martin Antonio; GSK Vaccine Institute of Global Health, particularly Danilo Moriel and Luisa Masai for the technical support and provision of SpyCEP, Slo, GAC, and SpyAD antigens for measuring IgG in Luminex 4-plex assay; the dedicated team of field and nursing staff, especially Sulayman Bah and Janko Camara, who delivered the study at MRC Unit The Gambia at LSHTM; Isatou Ndow for clinical trial organization; and the research support and clinical trials support offices at the Unit the Serum Institute of India for donating the vaccines for the study. Most importantly we thank the study participants and their parents who took part in the study.

***Financial support***. This work was supported by the Wellcome Trust in part by an Intermediate Clinical Fellowship (grant number 110058/Z/15/Z to T. I. d. S.) and a Clinical PhD Fellowship in Global Health (grant number 225467/Z/22/Z to A. J. K.).

Presented in part: Lancefield Symposium for Streptococcal and Streptococcal Diseases, Stockholm, 7–10 June 2022 and European Congress of Clinical Microbiology and Infectious Diseases, 9–12 July 2021, Online.

For the purpose of open access, the author has applied a CC BY public copyright licence to any Author Accepted Manuscript version arising from this submission.
