# Supplementary material for: Streptococcus pyogenes Colonization in Children Aged 24–59 Months in the Gambia: Impact of Live Attenuated Influenza Vaccine and Associated Serological Responses
Source: J Infect Dis. 2023 May 29;228(7):957–65. doi: 10.1093/infdis/jiad153 (PMC10547459; doi:10.1093/infdis/jiad153)
Supplement: jiad153_Supplementary_Data [file jiad153_supplementary_data.pdf]

Supplementary data

1. Details of *Streptococcus pyogenes* antigens for ELISA and Luminex

All recombinant proteins for ELISA were derived from the SF370 reference (M1/*emm*1/FCT-2) strain. Recombinant M1/Cpa/Mac(IdeS) were full length proteins, excluding N-terminal signal peptides and C-terminal cell wall anchor domains. Recombinant SpyCEP was a 61 kDa protein of the N-terminal region encompassing two of the three active sites without chemokine cleaving activity.[1]

All antigens for Luminex were donated by GSK Vaccines Institute for Global Health, and derived from SF370 reference strain. Recombinant protein antigens were detoxified SLO (amino acids 32–571), SpyAD (amino acids 27–849), and detoxified SpyCEP, (amino acids 34–1613).[2] GAC is the native carbohydrate with both polyrhamnose backbone and alternating N-acetylglucosamine at the side chain.[3]

Protein sequences for ELISA antigens:

> M1 48 kDa
MGNGDGNPREVIEDLAANNPAIQNIRLRYENKDLKARLENAMEVAGRDFKRAEELEKAKQ
ALEDQRKDLETKLKELQQDYDLAKESTSWDRQRLEKELEEKKEALELAIDQASRDYHRAT
ALEKELEEKKKALELAIDQASQDYNRANVLEKELETITREQEINRNLLGNAKLELDQLSS
EKEQLTIEKAKLEEEKQISDASRQSLRRDLDASREAKKQVEKDLANLTAELDKVKEDKQI
SDASRQGLRRDLDASREAKKQVEKDLANLTAELDKVKEEKQISDASRQGLRRDLDASREA
KKQVEKALEEANSKLAALEKLNKELEESKKLTEKEKAELQAKLEAEAKALKEQLAKQAEE
LAKLRAGKASDSQTPDTKPGNKAVPGKGQAPQAGTKPNQNKAPMKETKRQLEHHHHHH

>SpyCEP 61 kDa
MGDELSTMSEPTITNHAQQQAQHLTNTELSSAESKSQDTSQITLKTNREKEQSQDLVSEP
TTTELADTDAASMANTGSDATQKSASLPPVNTDVHDWVKTKGAWDKGYKGQGKVVAVIDT
GIDPAHQSMRISDVSTAKVKSKEDMLARQKAAGINYGSWINDKVVFAHNYVENSDNIKEN
QFEDFDEDWENFEFDAEAEPKAIKKHKIYRPQSTQAPKETVIKTEETDGSHDIDWTQTDD
DTKYESHGMHVTGIVAGNSKEAAATGERFLGIAPEAQVMFMRVFANDIMGSAESLFIKAI
EDAVALGADVINLSLGTANGAQLSGSKPLMEAIEKAKKAGVSVVVAAGNERVYGSDHDDP
LATNPDYGLVGSPSTGRTPTSVAAINSKWVIQRLMTVKELENRADLNHGKAIYSESVDFK
DIKDSLGYDKSHQFAYVKESTDAGYNAQDVKGKIALIERDPNKTYDEMIALAKKHGALGV
LIFNNKPGQSNRSMRLTANGMGIPSAFISHEFGKAMSQLNGNGTGSLEFDSVVSKAPSQK
GNEMNHFSNWGLTSDLEHHHHHH

>Cpa 77 kDa
MGKTVFGLVESSTPNAINPDSSSEYRWYGYESYVRGHPYYKQFRVAHDLRVNLEGSRSYQ
VYCFNLKKAFPLGSDSSVKKWYKKHDGISTKFEDYAMSPRITGDELNQKLRAVMYNGHPQ
NANGIMEGLEPLNAIRVTQEAVWYYSDNAPISNPDESFKRESESNLVSTSQLSLMRQALK
QLIDPNLATKMPKQVPDDFQLSIFESEDKGDKYNKGYQNLLSGGLVPTKPPTPGDPPMPP
NQPQTTSVLIRKYAIGDYSKLLEGATLQLTGDNVNSFQARVFSSNDIGERIELSDGTYTL
TELNSPAGYSIAEPITFKVEAGKVYTIIDGKQIENPNKEIVEPYSVEAYNDFEEFSVLTT
QNYAKFYYAKNKNGSSQVVYCFNADLKSPPDSEDGGKTMTPDFTTGEVKYTHIAGRDLFK
YTVKPRDTDPDTFLKHIKKVIEKGYREKGQAIEYSGLTETQLRAATQLAIYYFTDSAELD
KDKLKDYHGFGDMNDSTLAVAKILVEYAQDSNPPQLTDLDFFIPNNNKYQSLIGTQWHPE
DLVDIIRMEDKKEVIPVTHNLTLRKTVTGLAGDRTKDFHFEIELKNNKQELLSQTVKTDK

TNLEFKDGKATINLKHGESLTLQGLPEGYSYLVKETDSEGYKVKVNSQEVANATVSKTGI
TSDETLAFENNKEPLEHHHHHH

>Mac 36 kDa
MGSANQEIRYSEVTPYHVTSVWTKGVTPPANFTQGEDVFHAPYVANQGWYDITKTFNGKD
DLLCGAATAGNMLHWWFDQNKDQIKRYLEEHPEKQKINFNGEQMFDVKEAIDTKNHQLDS
KLFEYFKEKAFPYLSTKHLGVFPDHVIDMFINGYRLSLTNHGPTPVKEGSKDPRGGIFDA
VFTRGDQSKLLTSRHDFKEKNLKEISDLIKKELTEGKALGLSHTYANVRINHVINLWGAD
FDSNGNLKAIYVTDSDSNASIGMKKYFVGVNSAGKVAISAKEIKEDNIGAQVLGLFTLST
GQDSWNQTNLEHHHHHH

>SpyAD 89 kDa
MGDRASGETKASNTHDDSLPKPETIQEAKATIDAVEKTLSQQKAELTELATALTKTTAEINH
LKEQQDNEQKALTSAQEIYTNTLASSEETLLAQGAEHQRELTATETELHNAQADQHSKET
ALSEQKASISAETTRAQDLVEQVKTSEQNIAKLNAMISNPDAITKAAQTANDNTKALSSE
LEKAKADLENQKAKVKKQLTEELAAQKAALAEKEAELSRLKSSAPSTQDSIVGNNTMKAP
QGYPLEELKKLEASGYIGSASYNNYYKEHADQIIAKASPGNQLNQYQDIPADRNRFVDPD
NLTPEVQNELAQFAAHMINSVRRQLGLPPVTVTAGSQEFARLLSTSYKKTHGNTRPSFVY
GQPGVSGHYGVGPHDKTIIEDSAGASGLIRNDDNMYENIGAFNDVHTVNGIKRGIYDSIK
YMLFTDHLHGNTYGHAINFLRVDKHNPNAPVYLGFSTSNVGSLNEHFVMFPESNIANHQR
FNKTPIKAVGSTKDYAQRVGTVSDTIAAIKGKVSSLENRLSAIHQEADIMAAQAKVSQLQ
GKLASTLKQSDSLNLQVRQLNDTKGSLRTELLAAKAKQAQLEATRDQSLAKLASLKAALH
QTEALAEQAAARVTALVAKKAHLQYLRDFKLNPNRLQVIRERIDNTKQDLAKTTSSLLNA
QEALAALQAKQSSLEATIATTEHQLTLLKTLANEKEYRHLDEDIATVPDLQVAPPLTGVK
PLSYSKIDTTPLVQEMVKETKQLLEASARLAAENTSLVAEALVGQTSEMVASNAIVSKIT
SSITQPSSKTSYGSGSSTTSNLISDVDESTQRLEHHHHHH

Supplementary Figures and Tables

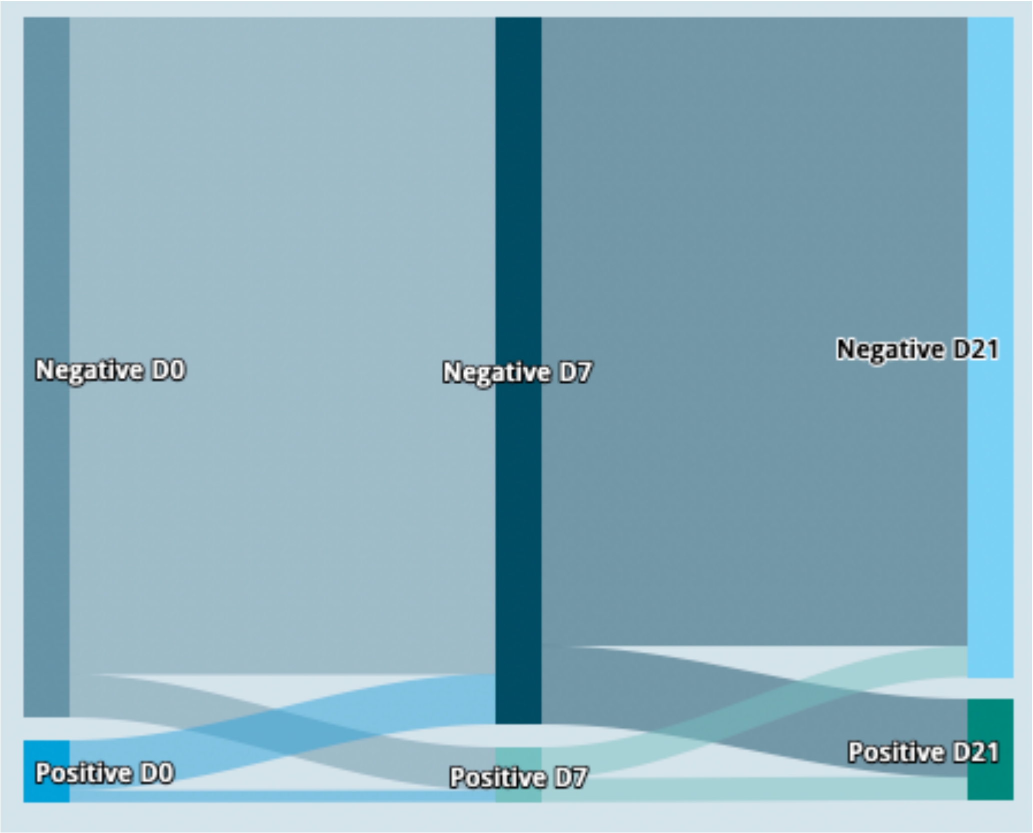

Figure S1: Colonization status over time. Sankey plot demonstrating the proportion of participants with positive and negative colonization status at each time point in the study. Colonization status determined by quantitative PCR targeting *speB*.

Table S1. *S. pyogenes* colonization during the study period. Colonization was determined by a *speB* real-time polymerase chain reaction at day 0, day 7 and day 21 of the study in children in the LAIV and control groups.

Time point	N	**Overall**, N = 320[1]	**LAIV**, N = 212[1]	**Control**, N = 108[1]	p-value[2]
**Baseline**	320				0.068
negative		294 (92%)	199 (94%)	95 (88%)	
positive		26 (8.1%)	13 (6.1%)	13 (12%)	
**Day 7**	320				0.7
negative		297 (93%)	196 (92%)	101 (94%)	
positive		23 (7.2%)	16 (7.5%)	7 (6.5%)	
**Day 21**	320				0.4
negative		277 (87%)	181 (85%)	96 (89%)	
positive		43 (13%)	31 (15%)	12 (11%)	
**Anytime**	320				0.8
negative		249 (78%)	164 (77%)	85 (79%)	
positive		71 (22%)	48 (23%)	23 (21%)	

[1] n (%)

[2] Pearson's Chi-squared test

Table S2. Factors associated with new colonization at D7 or D21 in the acquisition study group (n=294)

Characteristic	OR[1]	95% CI[1]	p-value
Age in months	0.99	0.96, 1.03	0.7
Intervention (Vaccinated)	1.92	0.93, 4.31	0.094
sex			
F	—	—	
M	1.34	0.69, 2.62	0.4
Positive respiratory virus at day 0	1.71	0.88, 3.28	0.11

[1] OR = Odds Ratio, CI = Confidence Interval

*p values for factors associated with new *Streptococcus pyogenes* acquisition within the acquisition study group (n=294) are derived from a logistic regression model with a composite outcome of colonization at either D7 or D21.

Table S3 Factors associated with *S. pyogenes* colonization prevalence in the entire cohort.

	OR	*95% CI*	*p-value*[1]
Intervention (vaccinated)	0.41	0.14-1.14	0.09
Day 21 (vs day 0)	0.88	0.34-2.28	0.8
Day 7 (vs day 0)	0.41	0.14-1.22	0.11
Positive respiratory virus at day 0	1.79	0.9-3.54	0.09
Age	0.98	0.94-1.02	0.29
Sex Male (vs female)	0.97	0.5-1.88	0.93
Intervention (vaccinated): Day 21	3.86	1.12-13.27	**0.03**
Intervention (vaccinated): Day 7	3.2	0.8-12.77	0.1

[1]p values for factors associated with *S. pyogenes* colonization are derived from a generalised logistic mixed-effect model, taking into account changes within individuals over time. Given the significant interaction between Intervention and Visit variables, the model was tested on both the vaccinated (intervention) and unvaccinated groups.

Table S4. Factors associated with *S. pyogenes* colonization prevalence in the unvaccinated group.

	OR	*95% CI*	*p-value*[1]
Day 21 (vs day 0)	0.86	0.3-2.51	0.79
Day 7 (vs day 0)	0.34	0.09-1.27	0.11
Positive respiratory virus at day 0	2.55	0.53-12.34	0.25
Age	1	0.92-1.1	0.95
Sex Male (vs female)	0.98	0.2-4.73	0.98

[1]p values for factors associated with *S. pyogenes* colonization in the live attenuated influenza vaccine group are derived from a generalised logistic mixed-effect model, taking into account changes within individuals over time.

Table S5A. Comparison of *S. pyogenes* density (log10 copies /mL) measured by qPCR in colonized participants (n=71) between vaccinated and unvaccinated groups at each time point of the study

	Study group		
	LAIV[1]	Control[1]	p-value[2]
D0	4.16 (1.10)	3.95 (0.89)	0.6
D7	4.43 (1.20)	5.24 (1.27)	0.2
D21	3.93 (0.84)	4.58 (1.27)	0.12

[1] Mean (SD)
[2] Welch Two Sample t-test

Table S5B. Comparison of *S. pyogenes* density (log10 copies /mL) measured by qPCR in episodes of colonization at D0 or D7 that were followed by persistent detection at the next episode (n=15) or not (n=31)

	Persistently positive episodes		
	No[1]	Yes[1]	p-value[2]
Colonization density	4.27 (1.23)	4.37 (1.01)	0.8

[1] Mean (SD)
[2] Welch Two Sample t-test

* Each PCR confirmed colonisation event at D0 and D7 was characterised as a "persistent episode" if at the subsequent study visit they remained positive.

Table S6. Comparison of symptoms reported by study participants by day 21 of the study in those who did or did not acquire *S. pyogenes* colonization.

	Acquisition status		
**Sypmtoms**	**No**, N = 249[1]	**Yes**, N = 45[1]	**p-value**[2]
fever	51 (20%)	12 (27%)	0.4
cough	94 (38%)	14 (31%)	0.4
runny nose	118 (47%)	26 (58%)	0.2
sore throat	1 (0.4%)	2 (4.4%)	0.062
skin sore	6 (2.4%)	4 (8.9%)	0.050

[1] n (%)

[2] Pearson's Chi-squared test; Fisher's exact test

Table S7. Comparison of age (in months) between colonized and non-colonized participants included in serological study (n=101).

Characteristic	colonised, N = 40[1]	non-colonised, N = 61[1]	p-value[2]
age	36 (29, 43)	32 (28, 39)	0.3

[1] Median (IQR)
[2] Wilcoxon rank sum test

Correlation between Luminex and ELISA for measurement of anti- *S. pyogenes* IgG antibodies

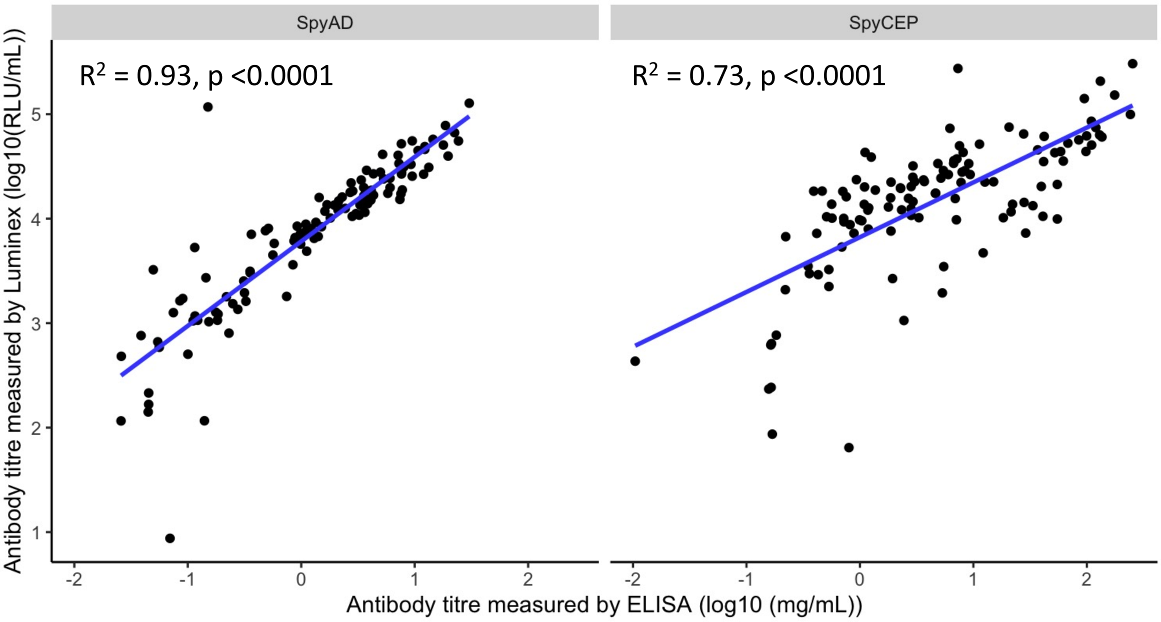

Figure S2: Correlation between IgG antibody titres measured from individual participants using ELISA and Luminex platforms. Log10 transformed IVIG-adjusted anti- protein activity for ELISA and Relative Luminex Units (RLU) for Luminex were analysed for correlation with Spearman method. Antigens used in the Luminex assay were obtained from National Institute of Biological Standards and Control, UK. Antigens in Luminex assay were obtained from GSK Vaccine institute for Global Health.

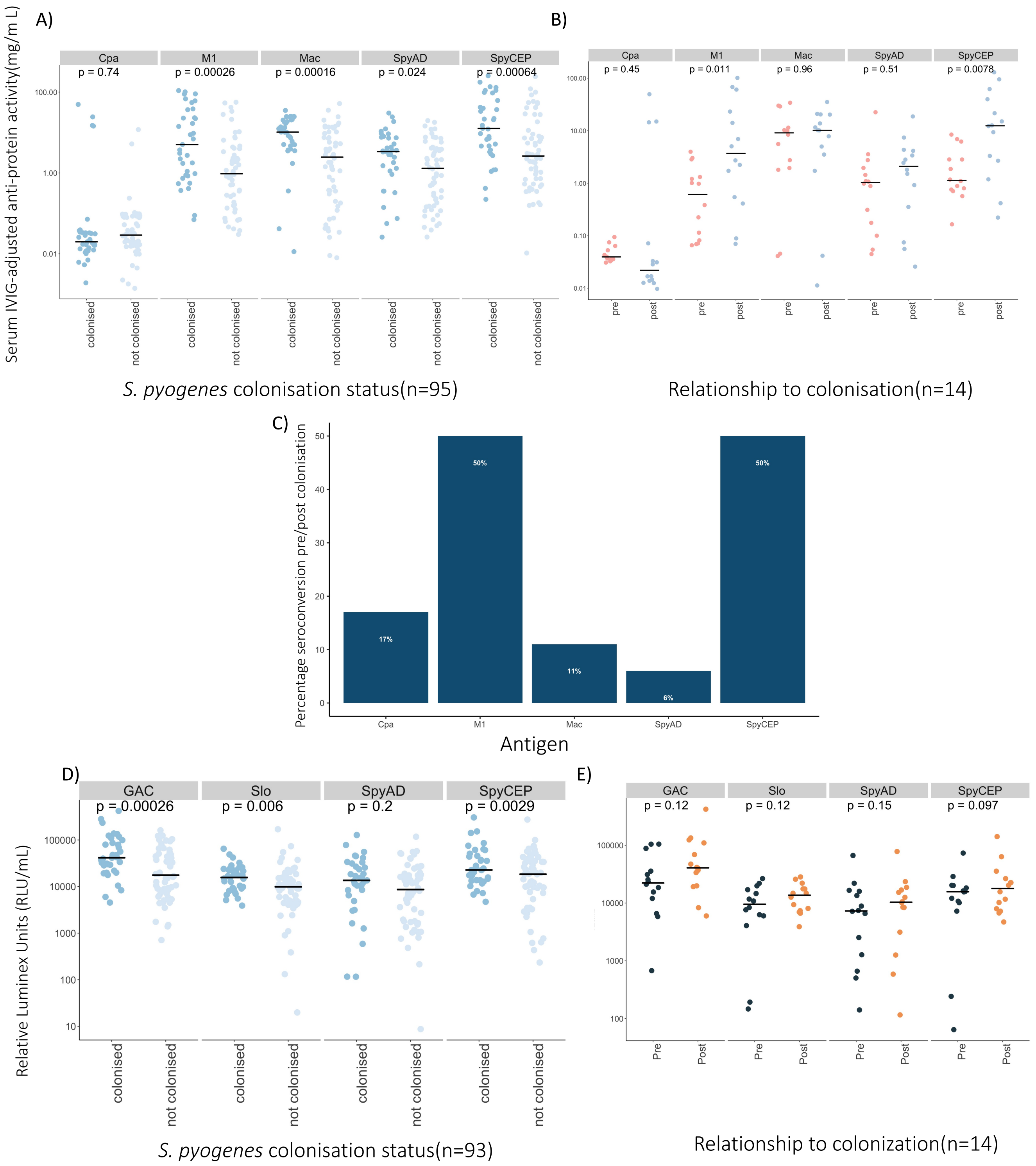

Figure S3. Serological responses to S. pyogenes colonization measured by ELISA and Luminex 4-plex, excluding 6 participants with sore throat (n=2) and/or infected skin sores (n=5) to ensure symptomatic pharyngitis or skin infection was not driving the serological responses observed in colonized children. A. Comparison of anti-protein IgG activity to Cpa, M1, Mac, SpyCEP, and SpyAD in participants (n=95) according to anytime S. pyogenes colonization status. **B.** Paired comparison of anti-protein IgG activity to Cpa, M1, Mac, SpyCEP, and SpyAD between day 0 and day 21 in newly colonized participants (n=14). Log10 transformed IVIG-adjusted anti-protein activity was compared with t-tests

(unpaired and paired respectively), horizontal line depicts the median value. **C**. Percentage of study participants (n=14) acquiring S. pyogenes during the study who seroconverted between day 0 and day 21. **D.** Comparison of IgG activity to GAC, SLO, SpyCEP, and SpyAD in participants (n=93) according to anytime S. pyogenes colonization status. **E**. Paired comparison of IgG activity to GAC, SLO, SpyCEP, and SpyAD between D0 and D21 in newly-colonized participants (n=14). Log_{10} transformed IVIG-adjusted anti-antigen activity was compared with t-tests (unpaired and paired respectively). Horizontal bar depicts the median value.

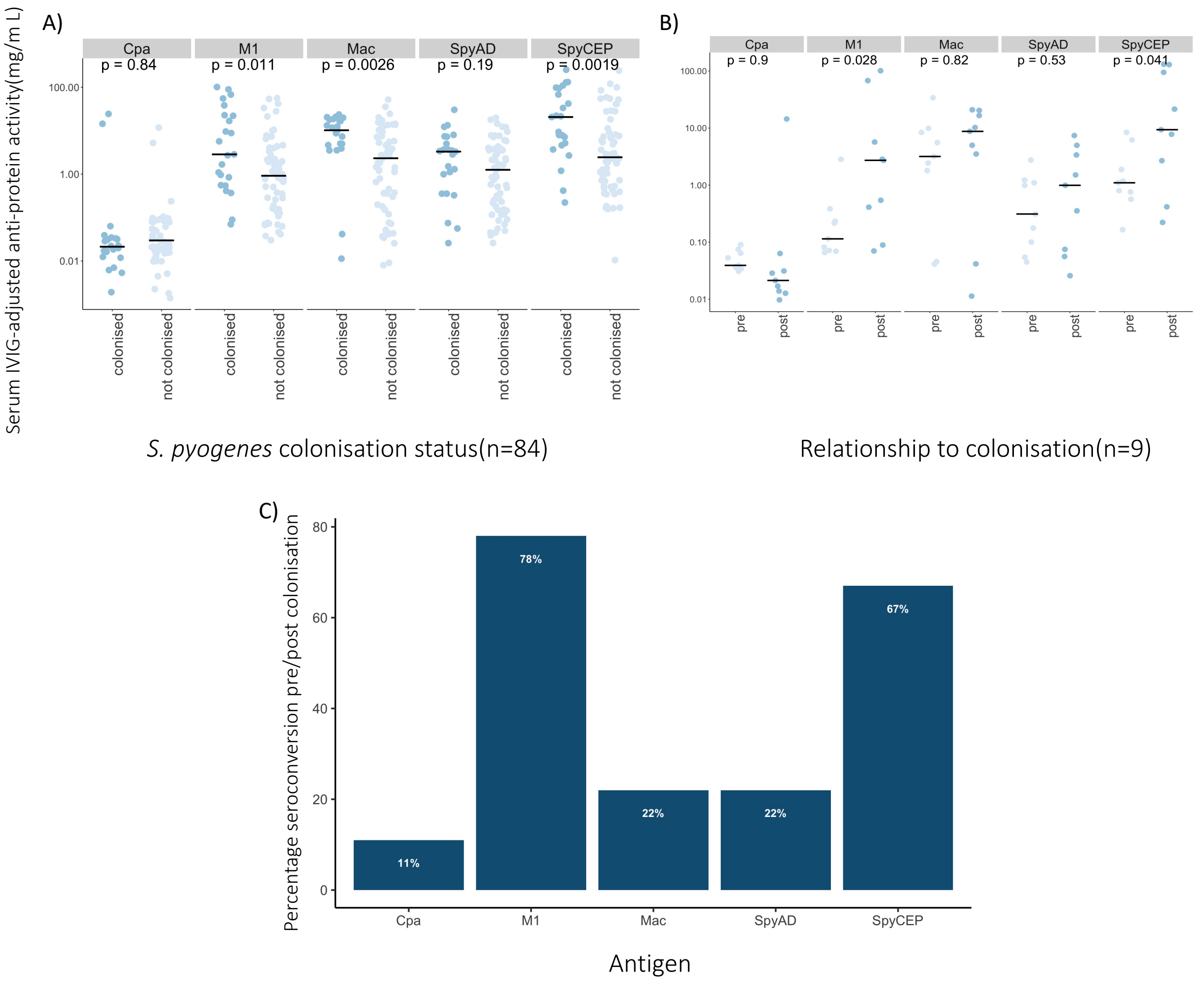

Figure S4. Serological responses to S. pyogenes colonization measured by ELISA excluding participants who were only colonized at D21, thereby attributing any serological responses to events occurring at least 14 days prior to serological measurement. **A.** Comparison of anti-protein IgG activity to Cpa, M1, Mac, SpyCEP, and SpyAD in participants (n=84) according to S. pyogenes colonization status on D0 or D7 of study. **B**. Paired comparison of anti-protein IgG activity to Cpa, M1, Mac, SpyCEP, and SpyAD between day 0 and day 21 in participants newly colonized (n=9) on D7 of study. Log_{10} transformed IVIG-adjusted anti-protein activity was compared with t-tests (unpaired and paired respectively), horizontal line depicts the median value. **C**. Percentage of study participants (n=18) acquiring S. pyogenes during the study who seroconverted between day 0 and day 21.

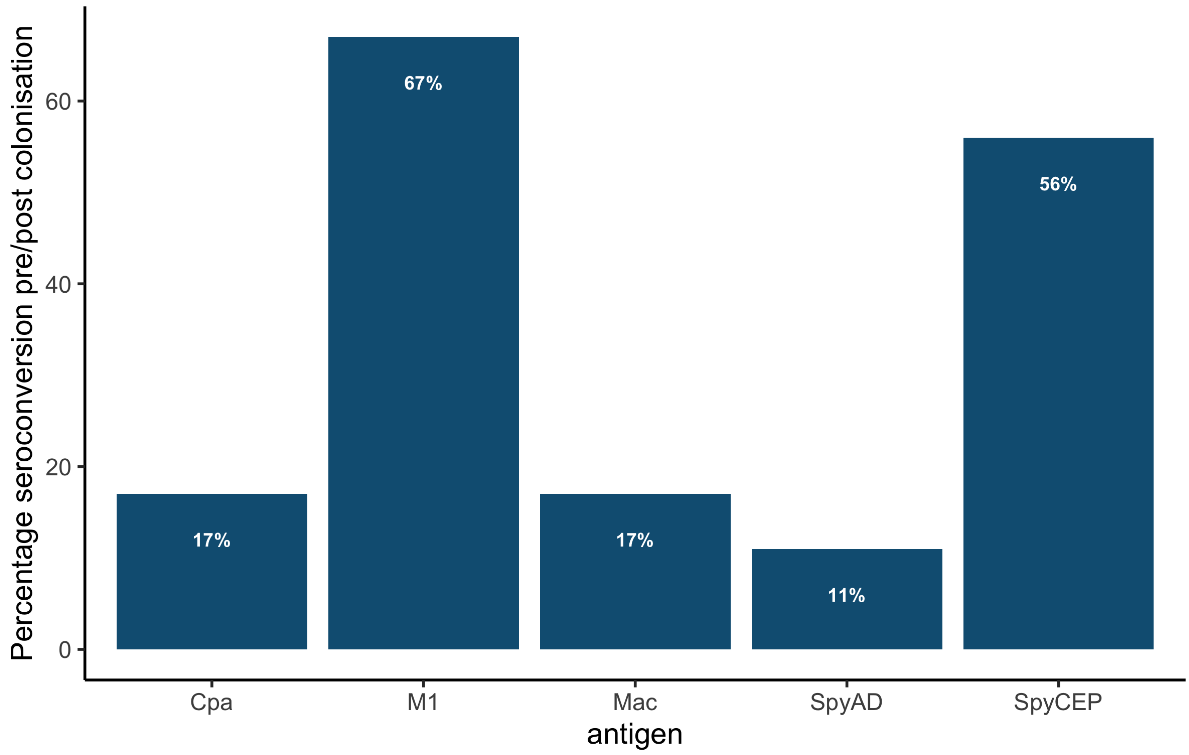

Figure S5: Percentage of newly-colonized study participants (n=18) who seroconverted between day 0 and day 21, using a more conservative definition of 4-fold increase in IgG titre measured by ELISA.

Supplementary references:

1. Shaw HA, Ozanne J, Burns K, Mawas F. Multicomponent Vaccines against Group A Streptococcus Can Effectively Target Broad Disease Presentations. Vaccines. Vol. 9, **2021**.
2. Bensi G, Mora M, Tuscano G, et al. Multi high-throughput approach for highly selective identification of vaccine candidates: the Group A Streptococcus case. Mol Cell Proteomics **2012**; 11:M111.015693.
3. Kabanova A, Margarit I, Berti F, et al. Evaluation of a Group A Streptococcus synthetic oligosaccharide as vaccine candidate. Vaccine **2010**; 29:104-14.
